# The Pupal Ectoparasitoid *Pachycrepoideus vindemmiae* Regulates Cellular and Humoral Immunity of Host *Drosophila melanogaster*

**DOI:** 10.3389/fphys.2019.01282

**Published:** 2019-10-11

**Authors:** Lei Yang, Bin Wan, Bei-Bei Wang, Ming-Ming Liu, Qi Fang, Qi-Sheng Song, Gong-Yin Ye

**Affiliations:** ^1^State Key Laboratory of Rice Biology and Ministry of Agriculture, Rural Affairs Key Laboratory of Molecular Biology of Crop Pathogens and Insects, Institute of Insect Sciences, Zhejiang University, Hangzhou, China; ^2^Division of Plant Sciences, College of Agriculture, Food and Natural Resources, University of Missouri, Columbia, MO, United States

**Keywords:** *Drosophila melanogaster*, *Pachycrepoideus vindemmiae*, ectoparasitoid, venom, cellular immunity, humoral immunity

## Abstract

The immunological interaction between *Drosophila melanogaster* and its larval parasitoids has been thoroughly investigated, however, little is known about the interaction between the host and its pupal parasitoids. *Pachycrepoideus vindemmiae*, a pupal ectoparasitoid of *D. melanogaster*, injects venom into its host while laying eggs on the puparium, which regulates host immunity and interrupts host development. To resist the invasion of parasitic wasps, various immune defense strategies have been developed in their hosts as a consequence of co-evolution. In this study, we mainly focused on the host immunomodulation by *P. vindemmiae* and thoroughly investigated cellular and humoral immune response, including cell adherence, cell viability, hemolymph melanization and the Toll, Imd, and JAK/STAT immune pathways. Our results indicated that venom had a significant inhibitory effect on lamellocyte adherence and induced plasmatocyte cell death. Venom injection and *in vitro* incubation strongly inhibited hemolymph melanization. More in-depth investigation revealed that the Toll and Imd immune pathways were immediately activated upon parasitization, followed by the JAK/STAT pathway, which was activated within the first 24 h post-parasitism. These regulatory effects were further validated by qPCR. Our present study manifested that *P. vindemmiae* regulated the cellular and humoral immune system of host *D. melanogaster* in many aspects. These findings lay the groundwork for studying the immunological interaction between *D. melanogaster* and its pupal parasitoid.

## Introduction

Parasitoids are unique venomous organisms among hymenopteran insects, with an estimated number of species ranging from 150,000 to 600,000 (Mrinalini and Werren, 2017). There is great potential for developing parasitoids as a crucial means of biological control. They lay eggs into the hemocoel (endoparasitoids) or on the surface (ectoparasitoids) of hosts (Moreau and Asgari, 2015). To ensure the survival and development of their offspring, parasitoids circumvent the host immune system with different adaptive strategies, namely, active immune suppression and passive immune evasion (Labrosse et al., 2003). Immune suppression is usually related to virulence factors, including venom (Asgari and Rivers, 2011), polydnaviruses (PDVs) (Gundersen-Rindal et al., 2013), virus-like particles (VLPs) (Grgacic and Anderson, 2006) and ovarian secretion (Mabiala-Moundoungou et al., 2010), and they work alone or cooperate with each other to regulate the cellular and humoral immune responses of hosts (Burke and Strand, 2014). In contrast, immune evasion occurs when wasp eggs are either covered with a fibrous layer or tightly adhered to host tissue, and the hosts fail to recognize it as non-self (Asgari et al., 1998; Eslin and Prévost, 2000; Hu et al., 2003). Both strategies are how parasitic wasps outwit their hosts.

Unlike mammals, insects lack acquired immune response, however, multiple innate defense responses have been highly developed to resist the invasion of parasitoids during the long-term arms race (Hoffmann, 1995). Insect innate immunity consists in cellular and humoral innate immunity (Lemaitre and Hoffmann, 2007). Cellular immunity response is mainly mediated by hemocytes. Much of our current understanding of hemocyte-mediated resistance to wasps lies in encapsulation. The *Drosophila* parasitoids are supposed to be valuable models for investigating their immunological interactions at the cellular immunity level (Carton et al., 2008). In general, *Drosophila melanogaster* recognizes parasitoids’ eggs as non-self once parasitoids lay eggs (Russo et al., 1996), followed by recruiting and spreading of plasmatocytes. Ultimately, lamellocytes collaborate with plasmatocytes to surround the wasp eggs and melanin is then deposited to seal it off, which is indispensable for killing the invaders (Williams, 2007). As a prominent humoral immune response of *Drosophila*, melanization plays an important role in fighting against parasitization (Tang, 2014). Prophenoloxidase (PPO) secreted by crystal cells and lamellocytes mainly contributes to the melanization of the wasp eggs (Dudzic et al., 2015). In addition to the melanization, the Toll, immune deficiency (Imd) and Janus kinase/signal transducer and activator of transcription (JAK/STAT) immune pathways associated with humoral immune responses are other exhaustively studied areas in confronting pathogenic infection and parasitization by parasitic wasps. In the Toll pathway, *Dif*/*Dorsal*, once activated, translocates into the nucleus and initiates the expression of antimicrobial peptides (AMPs) (Lehming et al., 1995; Nicolas et al., 1998; Wu and Anderson, 1998). In contrast, *caspase*-mediated N-terminal cleavage of *relish* activates the Imd pathway (Kim et al., 2006). Subsequently, N-terminal RHD-containing fragment (Rel-68) enters the nucleus, and a battery of AMPs are robustly produced (Stöven et al., 2000; Stoven et al., 2003). Unlike the complicacy of the Toll and Imd pathways, the JAK/STAT signaling pathway is more succinct, including three cytokine-like ligands named *unpaired* (upd) (Harrison et al., 1998), upd2 (Hombria et al., 2005), and upd3 (Agaisse et al., 2003; Wright et al., 2011), a transmembrane receptor, JAK and a Stat transcription factor (Rawlings et al., 2004; Myllymaki and Ramet, 2014). In *D. melanogaster*, an increasing number of studies have revealed that there is a connection between parasitization and the Toll, Imd and JAK/STAT pathways, while the regulatory effects vary greatly in different *Drosophila* parasitoid models (Wertheim et al., 2005; Martinson et al., 2014; Schmid, 2014; Yang et al., 2015; Louradour et al., 2017). Moreover, reactive oxygen species (ROS), a field of humoral innate immunity that has not been exploited fully, increased sharply in posterior signaling center (PSC) cells under parasitism, which conferred *Drosophila* resistance to wasp parasitism (Louradour et al., 2017). In short, they elaborately exploit cellular innate immunity and humoral innate immunity to cope with successful parasitism during the long-term antagonistic interaction between *Drosophila* and their parasitoid wasps.

Meanwhile, parasitoids have developed corresponding defense strategies to aid their progeny’s survival by virtue of various virulence factors. Venom, the fundamental parasitic factor either in endoparasitoids or ectoparasitoids, is the topic of interest in their host immune regulation. The primary functions of venom include inducing hosts paralysis, interrupting host development, suppressing the immunity of their hosts, etc (Coudron et al., 1990; Edwards et al., 2006; Tian et al., 2010; Kryukova et al., 2011). In a larval solitary endoparasitoid *Leptopilina boulardi*, long gland products induced a drastic decrease and an alteration of actin cytoskeleton in lamellocyte cells to inhibit the encapsulation (Labrosse et al., 2005a, b). Additionally, venom from *L. boulardi* inhibited the PO cascade of the *D. yakuba* larval hemolymph (Colinet et al., 2009). As a previously uncharacterized *Drosophila* parasitoid, *Ganaspis* sp.1 venom suppressed plasmatocyte calcium burst, resulting in its failure to migrate toward parasitoid eggs (Mortimer et al., 2013). There is some evidence indicating that secretions from the venom gland and ovary collaborate to regulate host physiology in *Asobara japonica* (Mabiala-Moundoungou et al., 2010). Furthermore, PDV is another thoroughly studied virulence factor, which is equally as important as venom in host immune regulation. In Braconidae and Ichneumonidae, Bracoviruses and Ichnoviruses imitated inhibitor kB (IkB) proteins of *Drosophila* to regulate immune NF-kappa B signaling by virtue of *ankyrins* (Bitra et al., 2012; Gueguen et al., 2013). In addition to the venom and PDVs, VLPs from *L. heterotoma* and *L. victoriae* also triggered immune suppression responses and further weakened the encapsulation phenotype in the host *Drosophila* (Morales et al., 2005; Heavner et al., 2017). In short, the mechanism of immunological interactions between parasitoids and their hosts is extremely complex and finely modulated.

*Pachycrepoideus vindemmiae* (Hymenoptera: Pteromalidae) is a versatile and solitary pupal ectoparasitoid of many flies whose hosts range from Drosophilidae to Anthomyiidae, Calliphoridae, Muscidae, Sarcophagidae, Tachinidae, Tephritidae, and so on (Marchiori and Borges, 2017). Unlike the multiple virulence factors in many koinobiont parasitoids, venom is the only required parasitic factor for successful parasitism of *P. vindemmiae.* Although there are many lines of research addressing the physiological mechanisms of immune modulation in the larval parasitoids of *Drosophila*, little has been investigated about the pupal parasitoids, let alone the research of the *P. vindemmiae*-*D. melanogaster* model. The main objective of the present study is to investigate the immune response of the host *D. melanogaster* once parasitized by *P. vindemmiae*. Our results demonstrated that great changes took place in cellular and humoral immunity of the host, including cell adherence, cell viability, hemolymph melanization and the Toll, Imd, and JAK/STAT immune pathways. Further studies illustrated the necessity of venom in the process of immune modulation. In brief, our present research opens a precedent for studying the pupal parasitoid-*Drosophila* system, which will contribute to a better understanding of the immunological interactions between the pupal parasitoids and their hosts.

## Materials and Methods

### Fly Strains

Host *D. melanogaster* stocks were raised on standard medium at 25°C with 60 ± 5% relative humidity and 16 h:8 h (light: dark) photoperiod. The stock w^1118^ originating from an indoor reared population was used as wild-type control. The following stocks were obtained from the Bloomington Stock Center (Indiana University, Bloomington, IL, United States): Dipt-lacZ, Drs-GFP (stock ID: 55707), 10^∗^Stat92E-GFP (stock ID: 26198), and Hop^Tum–1^ (stock ID: 8492).

### Parasitoid Collection and Rearing

The colony of *P. vindemmiae* was kindly provided by Prof. Yongyue Lu (South China Agricultural University, Guangzhou, China) in January 2016. Subsequently, *P. vindemmiae* was maintained with *D. melanogaster* pupae at 25°C with a photoperiod of 14 h: 10 h (light: dark) as previously described (Chen et al., 2015). Once closed, adults were held in glass containers and fed on 20% (v/v) honey solution.

### Cell Adherence Ability Assay

We could not separate pure hemocytes from *D. melanogaster* pupae considering that the bled fluids contained many fat granules. As a result, third instar larvae were used to obtain the hemocytes. The cuticle of Hop^Tum–1^ third instar larvae was gently pricked by forceps. Hemocytes from three larvae were bled on a glass slide containing 30 μl 10 mM phosphate buffer (PBS), pH 7.4 or different venom reservoir equivalents (VRE) dissolved in 30 μl PBS and allowed to adhere for 1 h. Thus, 1 VRE represents protein equivalents isolated from one venom reservoir and 2 VRE represents that of two venom reservoirs. Once adhered, it was easy to distinguish plasmatocytes and lamellocytes, which constituted the majority of all blood cells in Hop^Tum–1^ *Drosophlia* (Hanratty and Dearolf, 1993). Namely, plasmatocytes are small spherical cells and lamellocytes are large discoid cells. The adhered cells were washed three times with PBS, fixed with 3.7% paraformaldehyde solution (Sangon Biotech, Shanghai, China) for 15 min, and then washed three times with PBS before being permeabilized for 15 min with 0.1% Triton X-100 (Sangon Biotech, Shanghai, China). Subsequently, the fixed samples were incubated with 1% bovine serum albumin (BSA) for 30–60 min once washed three times with PBS. The F-actin was visualized by staining the cells with 1:1000 phalloidin-iFluor 488 (Abcam, Cambridge, United Kingdom) diluted in 1% BSA for 1 h. After this, the cells were washed 3 times with PBS and mounted by the SlowFade^TM^ Gold Antifade Mountant with DAPI (Life Technologies, Carlsbad, CA, United States). Photos were taken under white light and fluorescence view, respectively, by a Nikon eclipse TS-100 (Nikon, Japan). In the following analysis, the cell area of fluorescence staining larger than 400 μm^2^ was considered as lamellocytes.

### Hemocyte Viability Assay

Hemocytes were collected into the wells of a 96-well plate (Corning, New York, NY, United States) as mentioned above and then allowed to adhere for 1 h. Thirty microliter PBS, 1 VRE or Lysis Buffer (Promega, Madison, WI, United States) was added and incubated for 30 min. Cellular mortality was monitored by using the CellTox^TM^ Green Dye (Promega, Madison, WI, United States) according to the manufacturer’s instruction. Dead cells exhibited enhanced fluorescence as a result of the stable bond between dye and DNA, while no appreciable increase was observed in viable cells because of the integrity of the cell membrane. Photos were taken as mentioned above. The fluorescence value was measured with a setting of 485–510 nm as the excitation wavelength and 520–530 nm as the emission wavelength.

### Melanization Assay

For the melanization analysis *in vivo*, w^1118^ pupae within 12 h pupation were collected and then injected with 23 nl 1 mg/ml BSA, saturated phenylthiourea (PTU) or 0.5 VRE (half dilution of 1 VRE), 1 VRE or 2 VRE, respectively. Three hours later, pupae were inspected under a Leica DFC425 Camera attached to a stereomicroscope Leica M205 A (Leica, Wetzler, Germany). The melanization analysis was performed according to the previous study with some modifications (Gregorio et al., 2002). Briefly, about 15 *D. melanogaster* pupae (20 mg in total) were ground in liquid nitrogen and immediately suspended in 100 μl PBS, PTU or VRE dissolved in Tris buffer (100 mM, pH 7.2) followed by 16,000 g for 20 min centrifugation, and the supernatant was transferred to a new tube. After 30 min incubation at room temperature, 10 μl aliquot was mixed with the PO assay mixture prepared as previously described and subjected to assay by measuring the optical density (OD) at 520 nm (Gregorio et al., 2002).

### Fluorescence Microscopy and LacZ Activity Analysis

To determine the effects of parasitism on the Toll, Imd and JAK/STAT pathways, downstream transcription factors, AMPs or stress factors, including *drosomycin*, *diptericin, stat92E* and *thioester-containing protein 1(Tep1)*, were used as indicators. Hence, Dipt-lacZ; Drs-GFP stocks were used for both lacZ activity analysis and fluorescence microscopy. GFP detection was also conducted in 10^∗^Stat92E-GFP stock. Briefly, pupae were collected and parasitized for 1 h within 12 h after pupation. It was assumed that successful parasitism occurred when the envenomation lasted for more than 1 min, and the remaining that did not meet the criteria were removed. Pupae were reared as mentioned above. Then, pupae were photographed at 1, 6, 12, 24, 48, and 72 h after parasitization with GFP fluorescence channel using Nikon AZ100M (Nikon, Tokyo, Japan). Unparasitized *D. melanogaster* pupae were used as control.

LacZ activity was analyzed by quantifying the enzymatic activity of β-galactosidase as the previous study (Romeo and Lemaitre, 2008). In brief, five pupae were collected into a 2.0 ml Eppendorf tube. Then, 250 μl buffer A (60 mM Na_2_HPO_4_, 60 mM NaH_2_PO_4_, 10 mM KCl, 1 mM MgSO_4_, and 50 mM β-mercaptoethanol, pH 8.0) was added and homogenized for 30 s followed by supplementation of 250 μl buffer A and quickly vortexed. The mixture was centrifuged at 6000 × g for 5 min and the supernatant was transferred to a new 1.5 ml Eppendorf tube. Protein concentration was determined by a Modified Bradford Protein Assay Kit (Sangon Biotech, Shanghai, China) according to the manufacturer’s protocol. Finally, 30 μl aliquot was transferred to 96-well plates and 250 μl of 0.35 mg/ml O-nitrophenyl-β-D-galactoside dissolved in buffer A was added to each well followed by 37°C incubation. The β-galactosidase activity was determined at regular time intervals (10 min) by measuring the OD_420 nm_, and lacZ activity was calculated as Miller’s description: [(ΔOD_min_)/ΔT_min_]/protein concentration/0.0045 (Romeo and Lemaitre, 2008).

### RNA Extraction and Quantitative Real-Time PCR

Both parasitized and unparasitized w^1118^ pupae (5 each) were homogenized in 1 ml Trizol reagent (Invitrogen, Carlsbad, CA, United States). The total RNA was extracted according to the manufacturer’s protocol and cDNA was synthesized by using a PrimeScript^TM^ RT Reagent Kit with gDNA Eraser (Takara, Beijing, China). Quantitative RT-PCR (qPCR) was carried out using the TB Green^TM^ *Premix Ex Taq*^TM^ II (Tli RNaseH Plus) (Takara, Beijing, China) and run on a Bio-Rad CFX Connect (Bio-Rad, Hercules, CA, United States) instrument according to the manufacturer’s instructions. Relative expression levels of *drosomycin*, *diptericin* and *Tep1* were quantified and further normalized to reference gene *RPL32* (also referred as *R49*) using 2^–ΔΔCT^ method (Livak and Schmittgen, 2001). All the primers used were based on the previous study (Neyen et al., 2014).

### Data Analysis

The phalloidin staining area of hemocytes and the fluorescence intensity of pupal microscopy were measured by using image processing software, Image J 1.8.0 (Image J, NIH, United States). The total corrected fluorescence (TCF) of pupal microscopy was calculated as follows: integrated fluorescence density – (area of photograph) × (mean fluorescence of background). Data for two groups or more than three groups were analyzed by unpaired two-tailed student’s *t*-test and one-way analysis of variance (ANOVA) with Tukey’s test, respectively. In addition, a Chi-square test was conducted to test the difference in data of Supplementary Figure S1. There was statistical significance if *P* < 0.05. All statistical analyses were carried out using the data processing system (DPS) package version 9.5 (Tang and Zhang, 2013). All figures were plotted using GraphPad Prism 7.0 (GraphPad, San Diego, CA, United States).

## Results

### Effects of Venom on Cell Adherence

**FIGURE 1:**
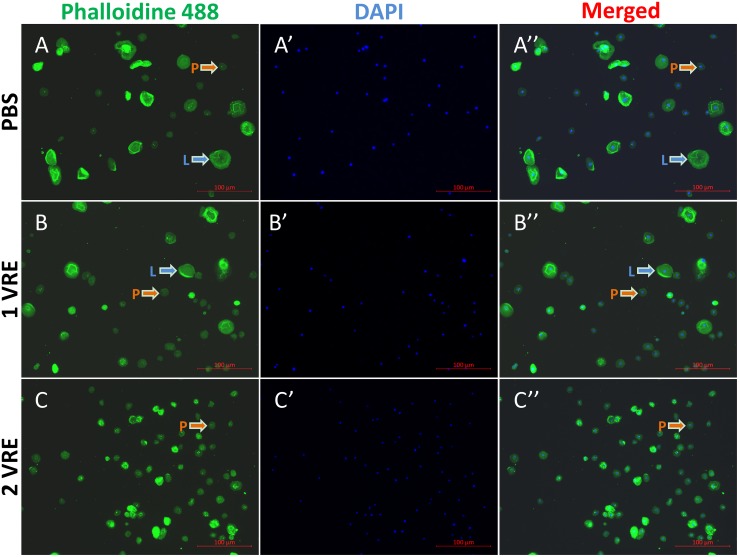
Immunofluorescence detection of hemocytes after venom treatment. The stained hemocytes incubated with PBS **(A–A′′)**, 1 VRE **(B–B′′)**, or 2 VRE **(C–C′′)** were photographed using fluorescence microscope. Subsequently, the staining area was counted. Plasmatocytes (P) and lamellocytes (L) are each indicated. Blue represents the nuclei stained with 4′, 6-diamidino-2-phenylindole (DAPI) and green represents the cytoskeleton marked with phalloidin 488.

As shown in Figure 1, plasmatocytes and lamellocytes adhered to the plate within 1 h after PBS treatment. The fluorescence area of 985 cells was measured and the results indicated that total hemocyte population comprised approximately 22% lamellocytes (Supplementary Figure S1). However, compared to the PBS control (Figure 1A), the ratio of lamellocytes was significantly decreased to 16% when treated with 1 VRE (Figure 1B). With the increasing dose of VRE, fewer adherent lamellocytes were observed, and the total number of lamellocytes declined to about 4% following 2 VRE incubation (Figure 1C). In contrast, low-concentration venom (0.67 VRE, 0.33 VRE and 0.17 VRE) had limited effects on lamellocyte adherence (data not shown).

### Effects of Venom on Cell Viability

**FIGURE 2:**
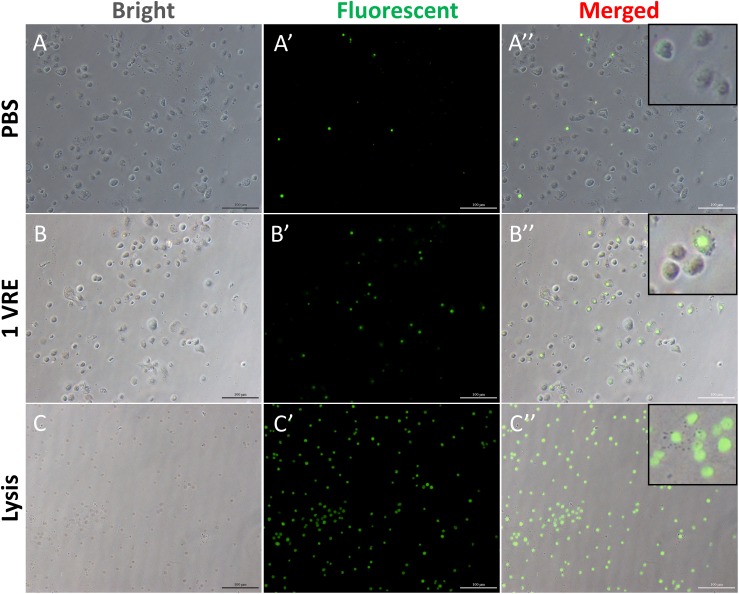
Cell viability assay after venom treatment. The hemocytes were incubated with PBS **(A–A′′)**, 1 VRE **(B–B′′)**, and lysate **(C–C′′)** followed by microscopic examination under white, fluorescent, and merged view, respectively. Fluorescently labeled cells represent dead cells.

To investigate whether the dysfunction in lamellocyte adherence was attributed to cell death, cell viability was detected using cellTox^TM^ green dye staining (Figure 2). In contrast to the considerably high survival rate under PBS treatment and abrupt decline in viable hemocytes under lysis treatment (Figures 2A,C), the percentage of GFP-positive cells was about twice as high as that in the PBS control after 1 VRE incubation (Figure 2B). Similar results were obtained when fluorescence value was measured, that is, crude venom induced a significant increase (2.12-fold) in fluorescence value (*P* < 0.05) (Supplementary Figure S2). Surprisingly, our findings indicated that GFP-positive cells were mainly occupied by plasmatocytes rather than lamellocytes in the presence of venom. Therefore, it was inferred that the functional disturbance of adherence in lamellocytes was unrelated to the cell death.

### Effects of Venom on Melaninization of the Hemolymph

**FIGURE 3:**
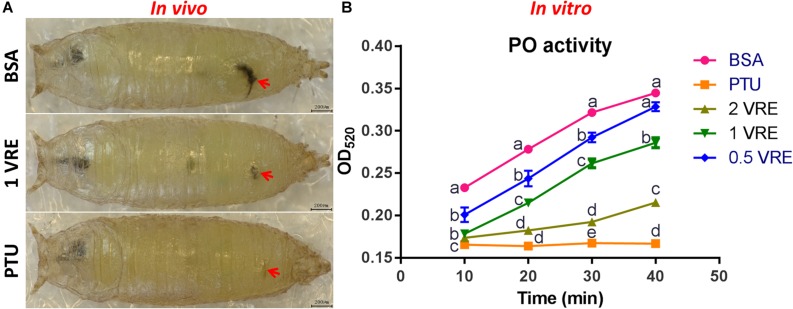
Effects of venom on melaninization of the host hemolymph. **(A)** Microscope inspection of w^1118^ pupae was performed 3 h after injection with BSA, 1 VRE, or PTU. **(B)** PO activity was assayed every 10 min by measuring the OD_520_ after hemolymph was co-incubated with BSA, PTU, or VRE. The results are shown as the mean ± standard error (*n* = 3); different lowercase letters above bars for the same time point indicate significant difference (*P* < 0.05).

To determine whether crude venom inhibited the melaninization of hemolymph, PO activity of pupal hemolymph was determined both *in vivo* and *in vitro*. As shown in Figure 3A, there was a slight melanization at the wound after 1 VRE injection compared to the strong melanization induced by BSA injection. In addition, quantitative analysis of PO activity was also conducted. Our results showed that 0.5, 1 and 2 VRE inhibited the melanization of the host hemolymph to different degrees (Figure 3B). Thus, 2 VRE significantly blocked the blackening of the hemolymph even after 40 min incubation. By comparison, a weaker inhibitory effect was observed in 1 VRE treatment. It appeared that 0.5 VRE had a small but significant effect on melanization after 10, 20, and 30 min incubation; however, the effect disappeared after 40 min incubation. These findings demonstrated that the inhibition of hemolymph melanization by crude venom was dose-dependent. Furthermore, these results shed light on the inhibitory effect of venom components on the host PO cascade.

### Effects of Parasitism on the Toll, Imd, and JAK/STAT Immune Pathways

**FIGURE 4:**
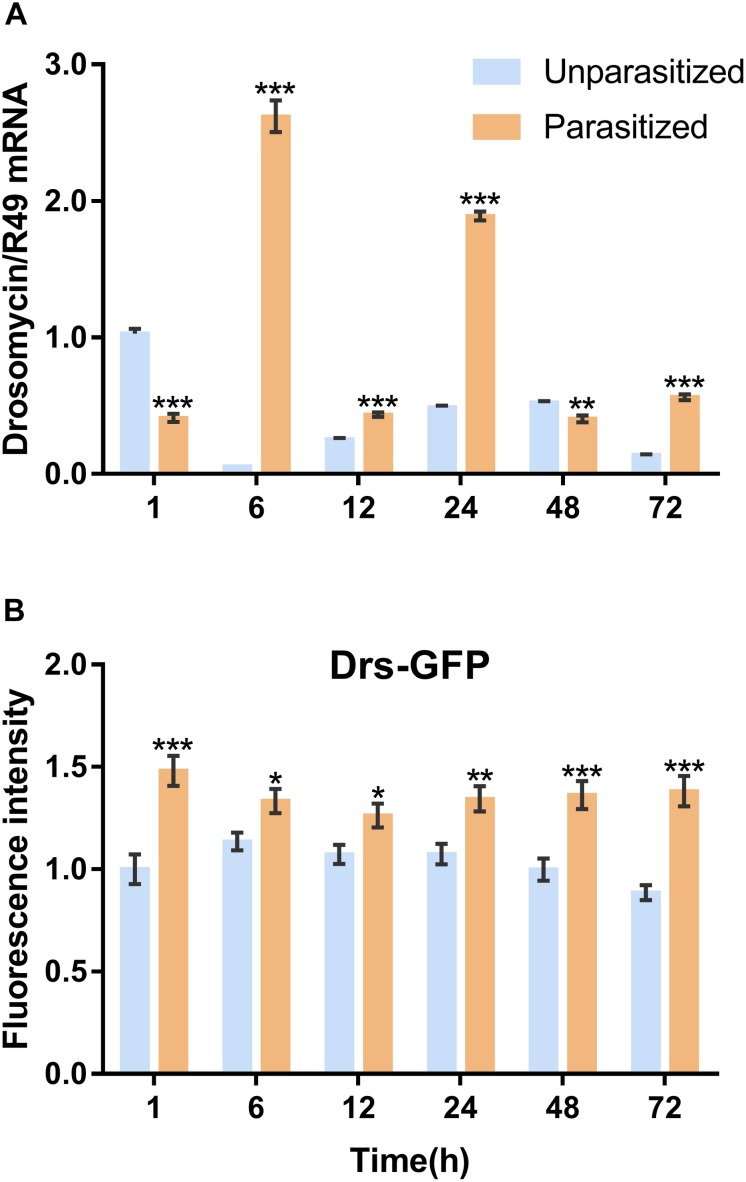
Effects of parasitism on the Toll pathway. **(A)** mRNA expression levels of *drosomycin* at 1, 6, 12, 24, 48, and 72 h both in parasitized and unparasitized *Drosophila* (*n* = 3), and ribosomal protein 49 (*R49*) was used as internal reference. **(B)** Fluorescence intensity measurement of drosomycin-GFP in parasitized and unparasitized *Drosophila* based on the time points mentioned above (*n* ≥ 10). The fluorescence intensity of 1 h unparasitized was normalized to 1.0. The results are shown as the mean ± standard error; *^∗^P* < 0.05, *^∗∗^P* < 0.01, and *^∗∗∗^P* < 0.001 compared to unparasitized.

**FIGURE 5:**
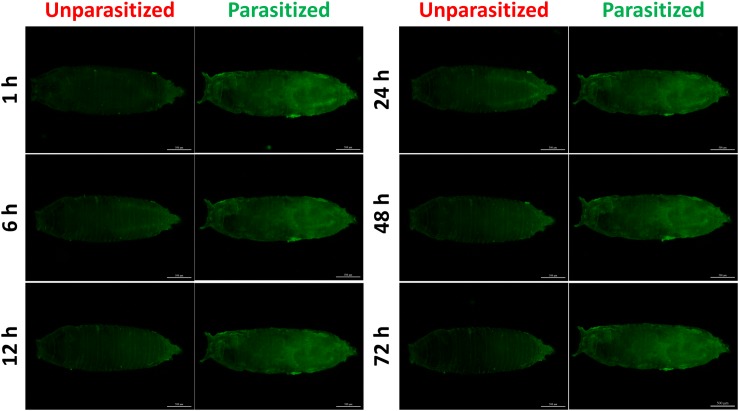
Fluorescence microscopy of unparasitized and parasitized drosomycin-GFP in *Drosophila* at different times. The green fluorescence was visualized through the cuticle using a florescence microscope.

To investigate the effect of parasitism on the Toll immune pathway, the expression level of *drosomycin*, a specific marker gene of the Toll immune pathways, was quantified. Results showed that the transcription levels of *drosomycin* increased sharply (46-fold) after 6 h of parasitism, and this effect lasted up to 72 h post-parasitism, although it was not shown at 48 h (Figure 4A). To further confirm the reliability of the results, protein expression level of *drosomycin* was quantified by measuring the fluorescence of drosomycin-GFP in *Drosophila* (Figure 4B). As Figure 5 showed, stronger fluorescence was generated on the parasitized *Drosophila* compared to unparasitized ones. Additionally, the fluorescence intensity was compared between parasitized and unparasitized *Drosophila*. Results showed that a higher level of *drosomycin* was induced both at the transcription level and the protein expression level once parasitized, whereas contrary circumstances happened at 48h post-parasitism (Figure 4). This unusual result might be explained by the smaller difference in qPCR results between unparasitized (0.52) and parasitized (0.40). Overall, it can be concluded that parasitism by *P. vindemmiae* activates the Toll pathway-dependent immune response.

**FIGURE 6:**
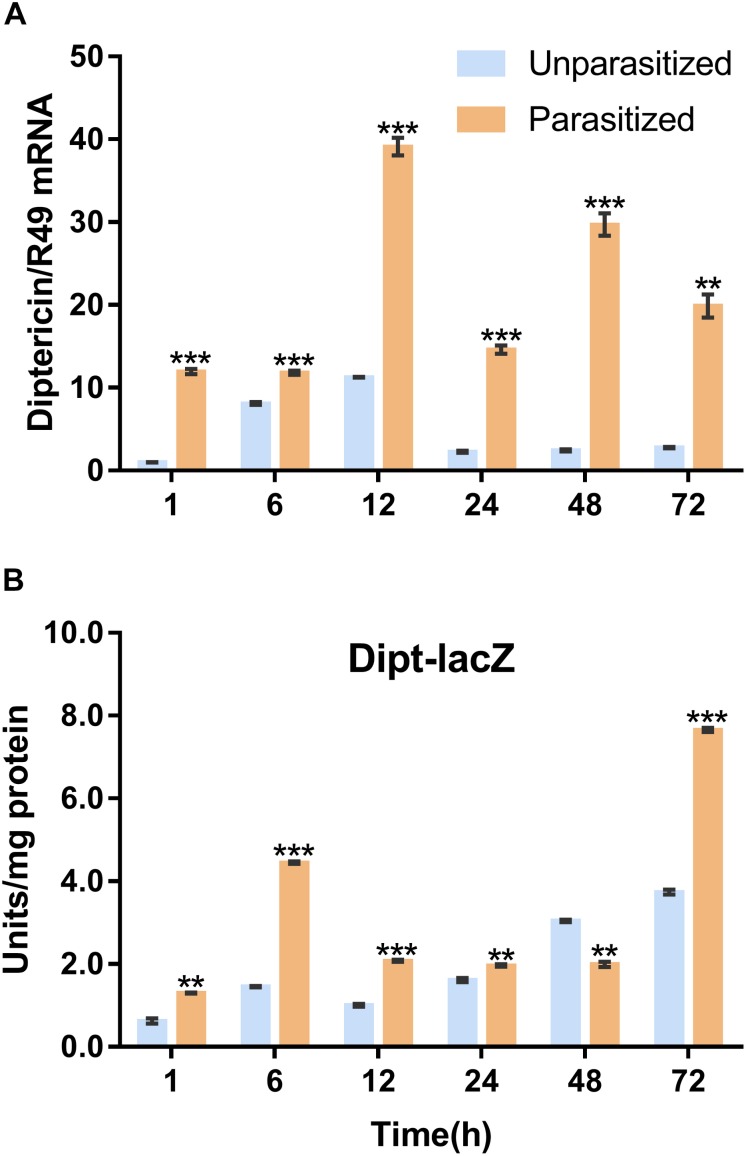
Effects of parasitism on the Imd pathway. **(A)** mRNA expression levels of *diptericin* at 1, 6, 12, 24, 48, and 72 h in parasitized and unparasitized *Drosophila* (*n* = 3). *R49* was used as internal reference. **(B)** Enzymatic activity assay of diptericin-lacZ both in parasitized and unparasitized *Drosophila* based on the time points mentioned above (*n* ≥ 10). The enzymatic activity of 1 h unparasitized was normalized to 1. The results are shown as the mean ± standard error; *^∗∗^P* < 0.01 and *^∗∗∗^P* < 0.001 compared to unparasitized.

Similarly, the Imd immune pathway is equally as important as the Toll pathway in immune defense response in *D. melanogaster*. Once parasitized by *P. vindemmiae*, the expression level of the Imd pathway specific marker gene *diptericin* was upregulated. As shown in Figure 6A, the mRNA transcriptional level of *diptericin* increased more than 10-fold at 1 h post-parasitism, and the high fold change lasted to 72 h compared with the unparasitized group. At the same time, lacZ enzymatic activity was basically consistent with qPCR results. As Figure 6B shows, the enzymatic activity of parasitized *Drosophila* was significantly higher than unparasitized both in the early and late stage of parasitism. However, the higher enzymatic activity was observed in unparasitized *Drosophila* at 48 h. Regardless of this point, parasitism contributed to the activation of the Imd immune pathway both at the transcription and the protein expression levels.

**FIGURE 7:**
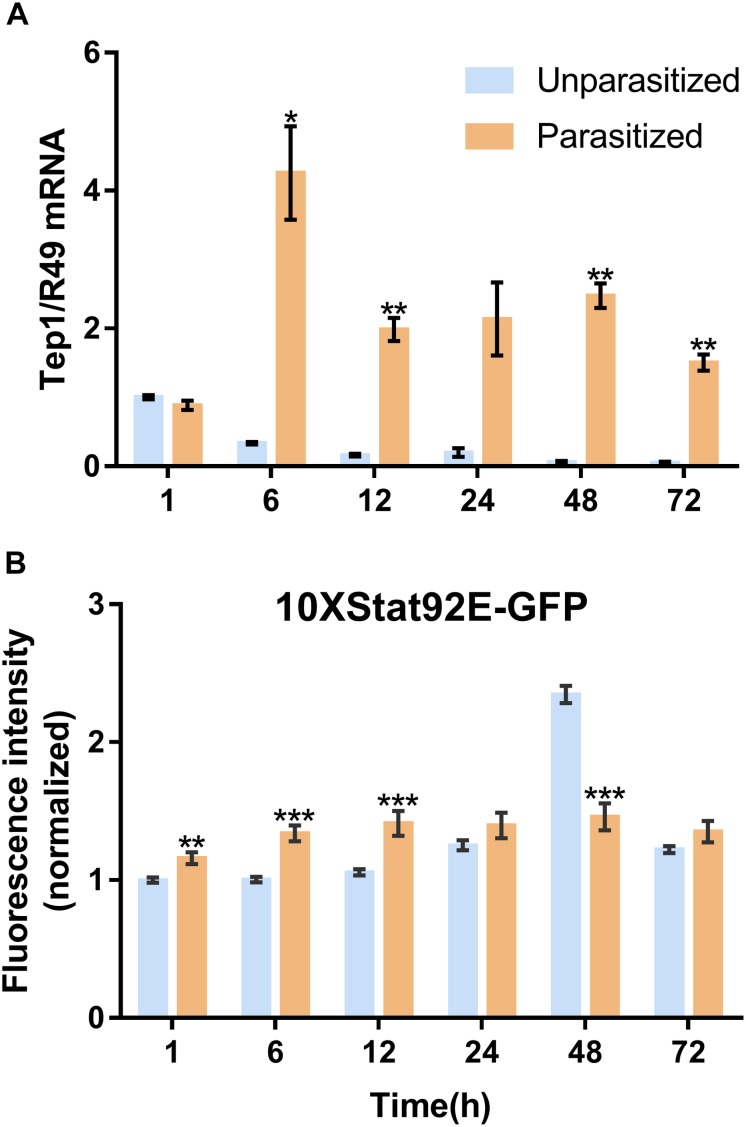
Effects of parasitism on the JAK/STAT pathway. **(A)** mRNA expression levels of *Tep1* at 1, 6, 12, 24, 48, and 72 h both in parasitized and unparasitized *Drosophila* (*n* = 3), and *R49* was used as internal reference. **(B)** Fluorescence intensity analysis of 10^∗^Stat92E-GFP *Drosophila* both in parasitized and unparasitized based on the time points mentioned above (*n* ≥ 10), and the fluorescence intensity of 1 h unparasitized was normalized to 1. The results are shown as the mean ± standard error; ^∗^*P* < 0.05, *^∗∗^P* < 0.01, and *^∗∗∗^P* < 0.001 compared to unparasitized.

**FIGURE 8:**
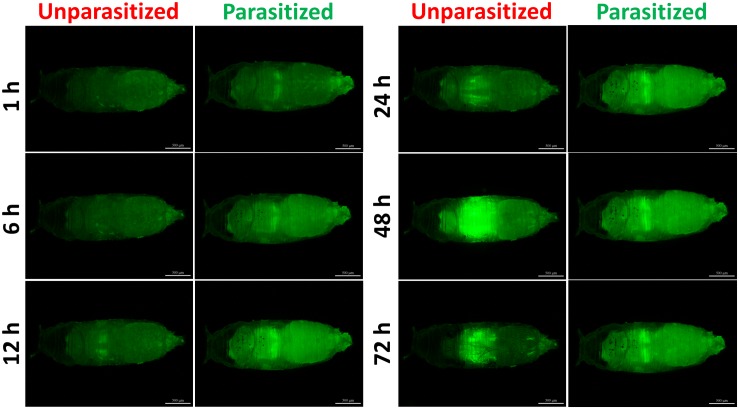
Fluorescence microscopy of unparasitized and parasitized 10^∗^Stat92E-GFP in *Drosophila* at different times. The green fluorescence was visualized through the cuticle using a florescence microscope.

Unlike the multifunctionality of the Toll and Imd immune pathways, the JAK/STAT immune pathway plays roles specifically in the process of immune responses against parasitoids and viruses. The Transcriptional level of *Tep1*, a marker gene of the JAK/STAT pathway, was detected. Similar with the previous results, the mRNA level of *Tep1* increased several times in *D. melanogaster* pupae once parasitized by *P. vindemmiae*, which lasted until the later stage of parasitization (Figure 7A). To clarify the facticity of the qPCR results, the expression levels of *Stat92E*, a transcription factor of *Tep1*, were further investigated by analyzing the fluorescence of 10^∗^Stat92E-GFP *Drosophila* both in unparasitized and parasitized pupae at 1, 6, 12, 24, 48, and 72 h. Experimental results indicated that there was a stronger GFP fluorescence signal at 1, 6, and 12 h in parasitized *Drosophila* (Figure 7B). However, no significant difference was shown at 24 h between the parasitized and unparasitized (Figure 7B). Conversely, as shown in Figure 8, a significant inhibitory effect on the activation of *Stat92E* at 48h post-parasitism was revealed, that is, weaker fluorescence intensity was recorded. These results indicated that the JAK/STAT immune pathway was activated during the early stage of parasitization.

## Discussion

The innate immune system of host insects involves cellular and humoral aspects. They orchestrate together to resist the successful parasitism of parasitic wasps. In *Drosophila*, hemocytes are the key players in cellular immunity response of *Drosophila*, which constitute the crucial adjective weapons in cellular immunity, especially lamellocytes, and are only produced by the immune system under parasitization or aberrant conditions (Rizki and Rizki, 1992). The precondition for encapsulation is that plasmatocytes are recruited and spread over the egg surface followed by adhesion of lamellocytes to the plasmatocyte-covered wasp eggs (Anderl, 2015). It has been reported that *A. japonica* venom significantly disturbed the spreading behavior of *D. melanogaster* hemocytes (Furihata et al., 2013). In a *L. Boulardi*/*D. melanogaster* model, virulent extracts of venom reservoir induced a drastic decrease in lamellocyte counts (Labrosse et al., 2005a). More research showed that more than 80% of plasmatocytes and granular cells from the host *Pseudoplusia includens* were unable to spread after oviposition by *Microplitis demolitor* within 2 h (Strand and Noda, 1991). In a closely related species, *Nasonia vitripennis*, the total number of plasmatocytes declined sharply after 60 min envenomation and further *in vitro* experiment proved that isolated crude venom indeed blocked adhesion and spreading of hemocytes (Rivers et al., 2002). In the present study, we primarily focused on alterations in lamellocyte adherence ability following crude venom treatment. Consistent with the above studies, *P. vindemmiae* crude venom exerted a similar inhibitory effect on adherence of lamellocytes as that of *N. vitripennis*, which disturbed normal lamellocyte function involved in encapsulation. In addition, the ratio of adhered lamellocytes gradually decreased with the increasing venom dosage. To investigate whether the disability of adherence in lamellocytes was caused by cell death, cell viability was evaluated. The results showed that cell death was indeed induced by venom but mainly on plasmatocytes. Hence, the underlying mechanism for inactivation in adhesion of lamellocytes is still unknown and further investigations are still needed.

Humoral immunity, equally important as cellular immunity, is common to all insects as well. As one of the prominent humoral immunity responses, melanization plays important roles in resisting microbial infection and parasitization. During melanization, phenols are oxidized to quinones followed by the formation of melanin (Tang, 2014). Successful parasitism is greatly determined by the dysfunction in hemolymph melanization. As a counter-immune strategy, venom proteins act in inhibiting the process of PO cascades in host insects. Several lines of research have identified venom proteins that are involved in the process, such as 50-kDa serine proteinase homolog (Vn50) (Asgari et al., 2003), Serpin (Colinet et al., 2009; Yan et al., 2017), Kazal-type serine protease inhibitors (Qian et al., 2015) and defensin-like peptide (Tian et al., 2010). Given that the immune response of *D. melanogaster* was successfully suppressed by venom, successful parasitism occurs and the wasp eggs hatch followed by larval feeding. This is a consequence of co-evolution in the arms race between parasitoids and their hosts. In this study, we thoroughly investigated the effects of the venom cocktail on the melanization of *Drosophila* pupae. First, *in vivo* injection indicated that venom significantly inhibited the blackening of the wound. Furthermore, in contrast to BSA treatment *in vitro*, hemolymph incubated with venom melanized to a lesser extent, and this inhibitory effect was gradually enhanced as the concentration increased from 0.5 VRE to 2 VRE, showing a dose-dependent effect. However, it is still a black box regarding how many venom proteins really function in this process, let alone their inhibitory mechanism.

As the core portion of humoral response, the Toll, Imd and JAK/STAT pathways play important roles against parasitism. For instance, the loss-of-function mutations of the JAK/STAT and Toll pathways in *Drosophila* larvae exhibited inadequate capacity to encapsulate the eggs of *L. boulardi* (Sorrentino et al., 2004). On the contrary, transgenic *Drosophila* of gain-of-function in the JAK/STAT or Toll pathway led to the plentiful formation of melanotic tumors (William and Jan, 1981; Gerttula et al., 1988; Harrison et al., 1995; Luo et al., 1995). In *L. boulardi*, the JAK/STAT signaling was significantly activated 27 h post-parasitization (Yang et al., 2015). Different from studies about the activation of the JAK/STAT and Toll pathways on parasitization, there have been limited studies about the crosslink between parasitization and the Imd immune pathway except for several lines of research based on high-throughput omics analysis. For instance, *L. heterotoma* venom specifically inhibited the Toll and Imd pathway signaling in fat body by microarray analysis (Schlenke et al., 2007). In contrast, genome-wide analysis indicated that transcription factor *Relish* and several AMPs downstream of the Imd pathway were strongly unregulated in response to *A. tabida* attack (Wertheim et al., 2005). In *N. vitripennis*, significant increases of several immune-related genes of the Toll and Imd pathways provided evidence that venom activated certain immune responses in envenomated hosts by whole gene expression profile analysis (Martinson et al., 2014). Thus, an 8.6-fold and 2.4-fold upregulation on *spätzle* and *relish* were shown, respectively. As a closely related species of *Nasonia*, it is inferred that parasitization may have similar effects on *Drosophila* immune signaling by *P. vindemmiae*. As expected, here we showed that the Toll and Imd immune pathways were activated by monitoring the transcription and expression of transcription factors and AMPs *in vivo* after parasitization. Similarly, our findings also indicated that the JAK/STAT pathway was activated during the early stage of parasitization, lagging behind the Toll and Imd pathways during this process. One unanticipated finding was that contrary circumstances occurred between the transcription level and the protein expression level at 48h post-parasitism. This observation could be due to the fact that pupae experienced a tissue regeneration process during this period. Separation of the pupal cuticle was initiated at 24 h after puparium formation and at around 48–50 h, the adult cuticle was formed (Carol et al., 1982). The transcript levels of a set of genes changed significantly during this process (Arbeitman et al., 2002). The latter point proposed by Wright et al. also found that a set of five L71 genes encoding polypeptides resembling AMPs were activated owing to the protection of pupal cuticle from bacterial infections in the late pupal stage (Wright et al., 1996). Therefore, their transcription or protein expression levels might not truly reflect the response. After 72 h in the unparasitized pupae, the eclosion of adults begins within the next few hours. Consequently, we did not monitor the transcription level and the protein expression level beyond 72 h. In fact, it is more complicated to evaluate the definite effect of parasitism on the Toll, Imd and JAK/STAT pathways. We postulate that venom plays major roles during this process.

**FIGURE 9:**
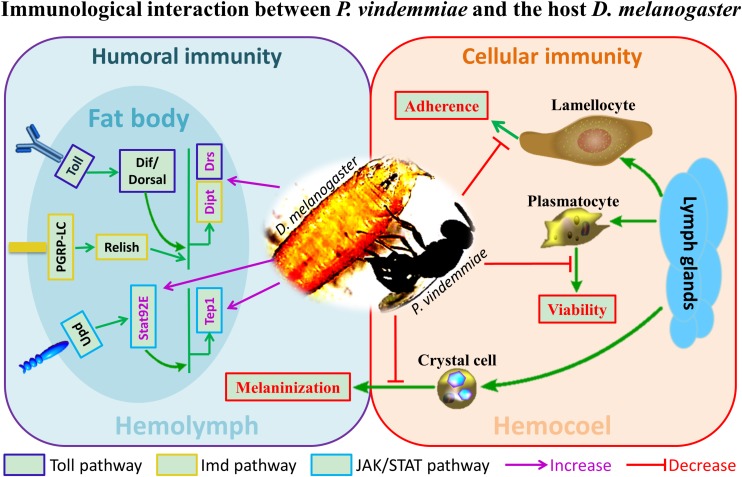
The schematic representation of immunological interaction between *P. vindemmiae* and the host *D. melanogaster.*

Previous studies on the regulation of *Drosophila* immune response by venom were mainly performed on larval endoparasitoids. In particular, the genera *Leptopilina*, *Ganaspis* and *Asobara* have been well studied in the context of immunology. Our findings provided preliminary information on the immunological interplay between *D. melanogaster* and its pupal ectoparasitoid *P. vindemmiae* for the first time (Figure 9). Based on the reported evidence, the importance of host immune regulation by ectoparasitoids could be summarized as follows. First, many immune-related proteins have been identified in ectoparasitoid venoms such as serine proteases and serine protease inhibitors (De Graaf et al., 2010; Zhu, 2016). Our unpublished results showed that immune-related proteins occupy the major categories of proteins in *P. vindemmiae* venom, a result consistent with these reports. It is reasonable then to believe that venom is important for ectoparasitoids to regulate the host immune response. In ectoparasitoid *S. guani*, venom was essential for the survival of the larvae to avoid host cellular immune attack, considering that they would contact with the hosts’ hemolymph released from the puncture wound (Li et al., 2018). Similarly, *P. vindemmiae* injected venom into the host hemocoel prior to laying eggs, and it is inferred that the persistently virulent effects on cellular defense of the host *Drosophila* also contributes to the successful development of their offspring, allowing the wasp offspring to feed more readily. More evidence revealed that envenomation by *N. vitripennis* induced significant increases in AMPs and their corresponding regulatory genes (Martinson et al., 2014), and our finding is consistent with the reported data. Based on this elaboration, we guess that the activation of the immune pathway of the host *Drosophila* is a preventative measure against bacterial or fungal infection and further enhances the nutritional quality of the host for larval feeding. In the host *Sarcophaga bullata*, melanization is an elaborative humoral immune response against foreign proteins such as parasitoid venom, and was inhibited by calreticulin from *N. vitripennis* venom (Siebert et al., 2015). Thus, it is speculated that the suppression of the host melanization is a coping strategy to avoid the dysfunction of venom for *P. vindemmiae*. Taken together, it is of vital importance for ectoparasitoid *P. vindemmiae* to regulate the host immune response. However, more research is still needed for understanding the underlying regulatory mechanisms.

As a groundbreaking research into the pupal ectoparasitoid of *D*. *melanogaster*, *P. vindemmiae* is attracting considerable interest in the field of biological control of fruit flies. Several attempts have been made on its potential for biological control in the last few years. One of the practical cases was that *P. vindemmiae* has prospects for control of olive fruit fly, *Bactrocera oleae* (Hoelmer et al., 2011). Additionally, a recent article reviewed that *Trichopria drosophilae*, a pupal parasitoid of *D. suzukii*, is desirable for high efficacy in biological control (Woltering et al., 2019). Similarly, as a well-known generalist pupal parasitoid of Diptera Cyclorrhapha, *P. vindemmiae* also possesses enormous potential on control of *D. suzukii* (Bonneau et al., 2019; Schlesener et al., 2019). To sum up, our explorative work on the crosstalk between ectoparasitoid *P. vindemmiae* and the host *Drosophila* will lay a foundation for providing new insights into biological control of fruit flies, and vastly propels the application of bio-control agent.

## Conclusion

The ways that host immune systems are regulated vary greatly in different host/parasitoid systems. Here, we proposed that venom of *P. vindemmiae* functioned as the crucial regulator in cellular and humoral immune signaling of the host *D. melanogaster*. Our test results showed that the decreased cell adhesion and viability, weakened hemolymph melanization and dysfunctions of the Toll, Imd and JAK/STAT pathways were associated with the high potency of venom. However, where the actual targets of venom lied is still unknown. The data presented in this study clearly advance the knowledge of the immunological interaction between *Drosophila* and its pupal ectoparasitoid *P. vindemmiae*.

## Data Availability Statement

All datasets generated for this study are included in the manuscript/Supplementary Files.

## Ethics Statement

We declare that appropriate ethical approval and licenses were obtained during our research.

## Author Contributions

LY, BW, and B-BW performed the experiments. LY, M-ML, and QF analyzed the data. LY, QF, Q-SS, and G-YY designed the experiments. LY, Q-SS, and G-YY wrote the manuscript. All authors gave final approval for publication.

## Conflict of Interest

The authors declare that the research was conducted in the absence of any commercial or financial relationships that could be construed as a potential conflict of interest.
