# Supplementary material for: The Pupal Ectoparasitoid Pachycrepoideus vindemmiae Regulates Cellular and Humoral Immunity of Host Drosophila melanogaster
Source: Front Physiol. 2019 Oct 11;10:1282. doi: 10.3389/fphys.2019.01282 (PMC6798170; doi:10.3389/fphys.2019.01282)
Supplement: Supplementary file 1 [file Data_Sheet_1.docx]

Supplementary Material


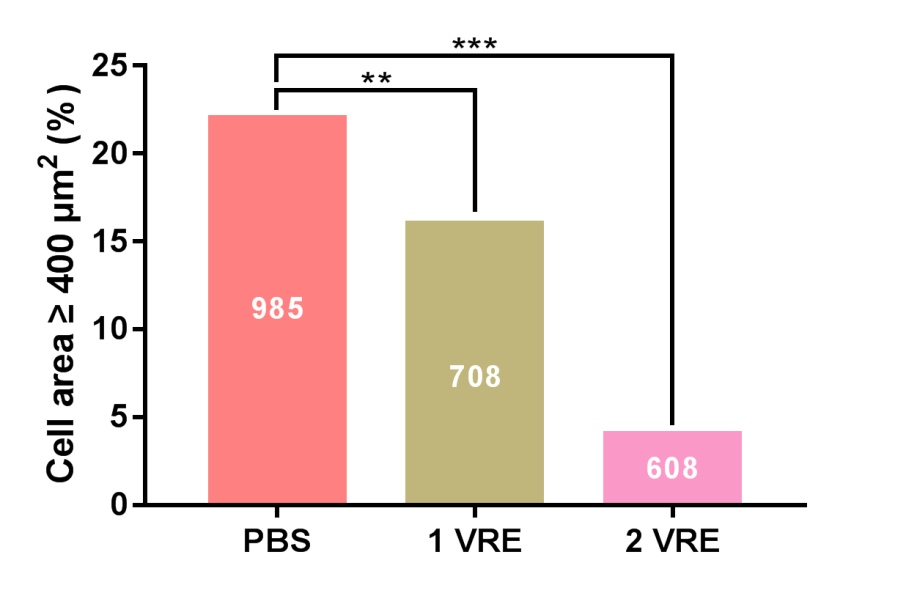


**Supplementary Figure 1.** Effects of venom on cell adherence. The ratio of lamellocytes was counted under PBS, 1 VRE or 2 VRE treatments following measuring the fluorescent area of 985, 708 and 608 stained cells, respectively. Significant difference was marked with “****” (*P* < 0.01) or “*****” (*P* < 0.001).


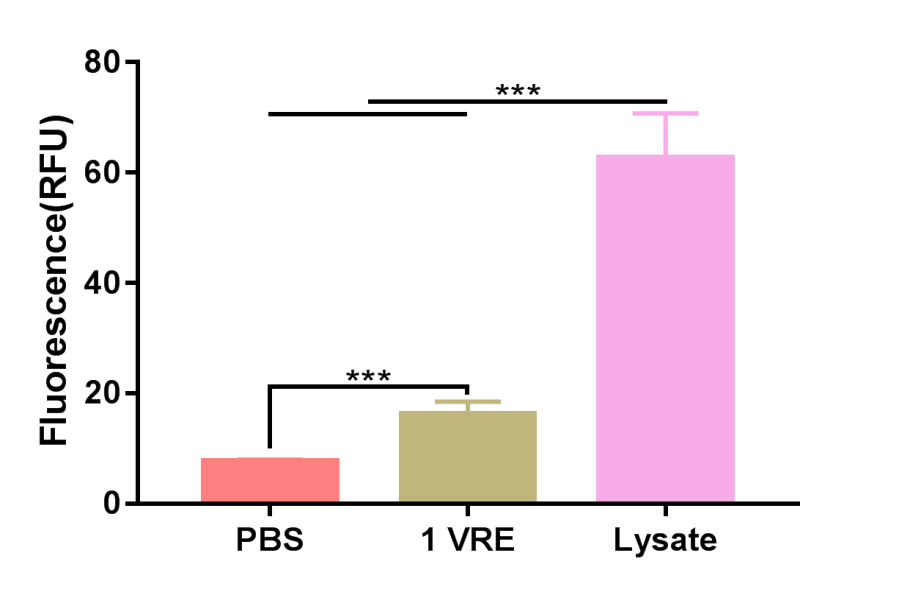


**Supplementary Figure 2.** Effects of venom on cell viability. Fluorescence value was measured under 485–500 nm_Ex_/520–530 nm_Em_ after being transferred to 96-well plates containing PBS, 1 VRE or lysate. The results are shown as the mean ± standard error (*n* = 3); “***” above bars indicate significant difference (*P* < 0.001).
